# Correlating Information Contents of Gene Ontology Terms to Infer Semantic Similarity of Gene Products

**DOI:** 10.1155/2014/891842

**Published:** 2014-05-22

**Authors:** Mingxin Gan

**Affiliations:** Dongling School of Economics and Management, University of Science and Technology Beijing, Beijing 100083, China

## Abstract

Successful applications of the gene ontology to the inference of functional relationships between gene products in recent years have raised the need for computational methods to automatically calculate semantic similarity between gene products based on semantic similarity of gene ontology terms. Nevertheless, existing methods, though having been widely used in a variety of applications, may significantly overestimate semantic similarity between genes that are actually not functionally related, thereby yielding misleading results in applications. To overcome this limitation, we propose to represent a gene product as a vector that is composed of information contents of gene ontology terms annotated for the gene product, and we suggest calculating similarity between two gene products as the relatedness of their corresponding vectors using three measures: Pearson's correlation coefficient, cosine similarity, and the Jaccard index. We focus on the biological process domain of the gene ontology and annotations of yeast proteins to study the effectiveness of the proposed measures. Results show that semantic similarity scores calculated using the proposed measures are more consistent with known biological knowledge than those derived using a list of existing methods, suggesting the effectiveness of our method in characterizing functional relationships between gene products.

## 1. Introduction

Over the last few years, domain ontologies have been successfully applied to describe entities within a variety of biological domains, with examples including the derivation of functional relationships between gene products based on the gene ontology (GO) [1–3], the inference of phenotype similarity between human diseases based on the human phenotype ontology (HPO) [4, 5], the modeling of general computational tasks in systems biology based on the systems biology ontology (SBO) [6], and many others [7–9]. With an ontology to provide controlled and structured vocabularies in a specific biological domain and annotations to characterize entities in the domain with the vocabularies, relationships between the entities can be quantified by their semantic similarities in the ontology, thereby providing a convenient yet powerful means of profiling the entities and their semantic relationships [1]. Nevertheless, the automated derivation of semantic similarity between entities based on their annotations in a domain specific ontology still remains a great challenge, appealing for the development of effective and convenient computational methods [10].

In general, a domain ontology provides a set of controlled and relational vocabularies for describing domain specific knowledge. The vocabularies, also referred to as concepts or terms, are often organized as a directed acyclic graph (DAG), in which vertices denote terms and edges represent semantic relationships between the terms. It is also common that an ontology has more than one semantic relationship. For example, in the gene ontology, there are multiple types of semantic relationships such as “*A* is_a *B*” (any instance of *A* is also an instance of *B*) and “*A* part_of *B*” (an instance of *A* is a component of some instances of *B*) [1]. Given such a domain specific ontology and annotations that map entities onto the terms, most existing methods first calculate pairwise semantic similarity between the terms using the structure of the ontology and annotations of entities and then derive similarity between the entities based on similarity between the terms [10–14].

Taking the gene ontology as an example, in order to achieve the former objective, Resnik proposed to use the information content (the negative logarithm of the relative frequency of occurrence of a term in annotations for a set of gene products) of the lowest common ancestor of two query terms to measure their semantic similarity [11]. Lin modified this measure by taking information contents of the query terms into consideration [12]. Schlicker et al. further incorporated the relative frequency of occurrence of the lowest common ancestor into the measure of Lin [14]. Jiang and Conrath proposed to incorporate the information contents of the query terms by using a formula different from that of Lin [13]. As another branch, Wang et al. proposed to calculate semantic similarity between GO terms using only the structural information of the underlying gene ontology, with the consideration of two types of semantic relationships: is_a and part_of [10].

With similarities between GO terms calculated, the semantic similarity between two query gene products was often calculated using a mean-max rule [10]. More specifically, given a single GO term and a collection of GO terms, the similarity between the term and the collection was defined as the maximum similarity between the term and every term in the collection. Furthermore, the similarity between two collections of GO terms was defined as the average of similarity between every term in a collection and the other collections. Finally, since a gene product was annotated by a collection of GO terms, semantic similarity between two gene products was defined as the similarity between the corresponding two sets of GO terms.

The above methods have been successfully applied to a variety of fields, with examples including the calculation of functional similarity between proteins based on the gene ontology (GO) for the inference of disease genes [2], the characterization of phenotype similarity between human diseases based on the human phenotype ontology (HPO) [5], and many others [7]. Software packages implementing these methods have also been released and publically available in the community of bioinformatics and computational biology, with examples including GOSemSim [15], FuSSiMeG [16], and OWLSim [4]. However, disadvantages of these methods are also obvious. For example, although methods such as those in [12–14] took efforts to modify the method of Resnik [11], their methods often performed worse than that of Resnik in real applications [10], suggesting that the revision of information contents can hardly be effective. Also, although Wang et al. systematically considered the structure and multiple semantic relationships of the gene ontology [10], they discarded the valuable resource of information contents of GO terms, resulting in a method performing worse than that of Resnik in many applications such as the prioritization of candidate genes [2]. In addition, as we shall see in the Results section, all of these methods tend to overestimate similarity between proteins that are actually not similar in their functions, thereby yielding misleading results in applications.

With these understandings, we propose in this paper to represent a gene product using a vector that is composed of information contents of GO terms annotated for the product in the gene ontology. Based on this notion, we suggest calculating semantic similarity between gene products as the relatedness of their corresponding vectors using three measures: Pearson's correlation coefficient, cosine similarity, and the Jaccard index. We focus on the biological process namespace of the gene ontology and annotations of proteins of the budding yeast* Saccharomyces cerevisiae* to perform a series of comprehensive studies on the effectiveness of the proposed measures. We calculate semantic similarity scores between yeast genes relying on the biological process domain of the gene ontology, use the resulting semantic similarity scores to measure functional relationships between the proteins, and study the consistency between such relationships and known biological knowledge. Results on 141 yeast biochemical pathways, 1,022 protein families, and two large-scale yeast protein-protein interaction networks show that semantic similarity scores calculated using the proposed measures are more consistent with biological knowledge than those derived using a list of existing methods, suggesting the effectiveness of our method in characterizing semantic similarity between gene products.

## 2. Methods

### 2.1. The Gene Ontology and Species Specific Annotations

The gene ontology (GO) provides a controlled vocabulary of terms for describing characteristics of gene products. This ontology covers three domains: biological process (BP), molecular function (MF), and cellular component (CC). The biological process domain defines operations or sets of molecular events with a defined beginning and end, pertinent to the functioning of living cells, tissues, organs, and organisms. The molecular function domain represents the elemental activities of a gene product at the molecular level, such as binding or catalysis. The cellular component domain describes the parts of a cell or its extracellular environment [1]. Each of these three domains is organized according to a directed acyclic graph (DAG) structure, represented as *G* = (*V*, *E*), where *V* is a set of vertices denoting concepts and *E* is a set of edges denoting semantic relationships between the terms. In such a graph, we use *P*_*t*_ and *C*_*t*_ to denote the sets of parents and children of term *t*, including *t* itself, respectively, and we use *A*_*t*_ and *D*_*t*_ to denote ancestors and descendants of term *t*, including *t* itself, respectively. Note that in the gene ontology, there are multiple types of semantic relationships such as “*A* is_a *B*” (any instance of *A* is also an instance of *B*) and “*A* part_of *B*” (an instance of *A* is a component of some instance of *B*).

A species specified annotation provides a mapping from a gene product of the species to a term in a domain (BP, MF, or CC) of the gene ontology. Following common specifications, the annotation of a gene product with term *t* implies the annotation of the gene product with all ancestors of *t*. With this notion, we represent annotations of gene product *g* using a binary annotation vector **a**_*g*_ = (*a*_*gi*_)_|*V*|×1_, where *a*_*gi*_ = 1 if *g* is annotated by the term indexed by *i* or its descendants and |*V*| the total number of terms in a domain.

## 3. Semantic Similarity as Correlation of Information Contents

Given a domain of the gene ontology and annotations for a set of gene products, the probability that a product annotated by term *t* or its descendants is estimated using the relative frequency of occurrence of term *t* and its descendants in the annotations is calculated by

(1) $$\begin{array}{c} \text{Pr}\left(t\right)=\frac{1}{N}\underset{i\in D_{t}}{\sum }n_{i}, \end{array}$$

where *n*_*i*_ is the number of annotations with term *i* and *N* the total number of annotations. The information content of term *t* is then calculated as

(2) $$\begin{array}{c} \text{IC}\left(t\right)=-\log\;\text{Pr}\left(t\right). \end{array}$$

Moreover, information contents of all terms in the domain can be represented as a vector **q** = (*q*_*i*_)_|*V*|×1_ with *q*_*i*_ being the information content of the term indexed by *i*. Calculating the Hadamard (entrywise) product of **a**_*g*_ and **q**, we obtain the vector of information contents for gene product *g* as **x**_*g*_ = **q**∘**a**_*g*_ = (*x*_*gi*_)_|*V*|×1_, where *x*_*gi*_ = *q*_*i*_ × *a*_*gi*_ for *i* = 1,…, |*V*|. With such a vector calculated for every gene product, we propose the following three measures to quantify semantic similarity between two entities.

First, we propose to calculate the similarity as the absolute value of Pearson's correlation coefficient between the two vectors **x**_*g*_ and **x**_*h*_ for two gene products *g* and *h* as

(3) $$\begin{array}{c} S_{gh}^{\left(\text{correlation}\right)}=\left|\frac{\sum_{1\leq i\leq |V|}\left(x_{gi}-{\overset{-}{x}}_{g}\right)\left(x_{hi}-{\overset{-}{x}}_{g}\right)}{\sqrt{\sum_{1\leq i\leq |V|}{\left(x_{gi}-{\overset{-}{x}}_{g}\right)}^{2}}\sqrt{\sum_{1\leq i\leq |V|}{\left(x_{hi}-{\overset{-}{x}}_{h}\right)}^{2}}}\right|. \end{array}$$

In this measure, we assume that information contents for the two gene products, **x**_*g*_ and **x**_*h*_, have a linear relationship, say,

(4) $$\begin{array}{c} x_{g}=\alpha +\beta x_{h}. \end{array}$$

Hence, it is natural to use the coefficient of determination (*r* ^2^) that measures how good the observations fit this linear model to quantify the similarity between the two vectors. To ease the computation, we simply calculate the absolute value of the correlation coefficient instead of *r* ^2^. Note that exchanging **x**_*g*_ and **x**_*h*_ in the linear model yields the same *r* ^2^.

Second, we calculate the similarity as the cosine of the angle between the two vectors **x**_*g*_ and **x**_*h*_ for two gene products *g* and *h* as

(5) $$\begin{array}{c} S_{gh}^{\left(\text{cosine}\right)}=\frac{\sum_{1\leq i\leq |V|}x_{gi}x_{hi}}{\sqrt{\sum_{1\leq i\leq |V|}x_{gi}^{2}}\sqrt{\sum_{1\leq i\leq |V|}x_{hi}^{2}}}. \end{array}$$

This is equivalent to calculating the uncentered correlation coefficient of the two vectors. It is evident that the cosine measure will yield similar results as those of the correlation measure when the means of **x**_*g*_ and **x**_*h*_ are small.

Third, we calculate the similarity as the Jaccard index of the two annotation vectors **a**_*g*_ and **a**_*h*_ for two gene products *g* and *h* as

(6) $$\begin{array}{c} S_{gh}^{\left(\text{Jaccard}\right)}=\frac{\sum_{1\leq i\leq |V|}\left(a_{gi}\wedge a_{hi}\right)}{\sum_{1\leq i\leq |V|}\left(a_{gi}\vee a_{hi}\right)}. \end{array}$$

This is equivalent to calculating the ratio of the number of elements in the intersection and union of the two annotation sets for gene products *g* and *h*.

## 4. Existing Methods for Calculating Semantic Similarity

Most existing methods first derive similarity scores between terms and then calculate semantic similarity scores between gene products as similarity scores between collections of annotated terms for the products. More precisely, there have been two main categories of methods for calculating pairwise concept similarity scores: (1) approaches based on information contents of terms in the gene ontology and (2) methods based on the structure of the gent ontology.

The first group of approaches calculates similarity between two terms *u* and *v* relying on the information content of the most specific term *m*_*uv*_ in their common ancestors. Generally, a term with more specific meaning tends to have a higher information content and hence

(7) $$\begin{array}{c} m_{uv}=\arg\underset{w\in A_{u}\cap A_{v}}{\max}\text{IC}\left(w\right). \end{array}$$

With this notion, Resnik [11] defined the similarity between *u* and *v* as

(8) $$\begin{array}{c} T_{uv}^{\left(\text{Resnik}\right)}=\text{IC}\left(m_{uv}\right)=-\log\;\text{Pr}\left(m_{uv}\right). \end{array}$$

Lin [12] defined the similarity as

(9) $$\begin{array}{c} T_{uv}^{\left(\text{Lin}\right)}=\frac{2\log\;\text{Pr}\left(m_{uv}\right)}{\log\;\text{Pr}\left(u\right)+\log\;\text{Pr}\left(u\right)}. \end{array}$$

Schlicker et al. [14] define the similarity as

(10) $$\begin{array}{c} T_{uv}^{\left(\text{Schlicker}\right)}=\frac{2\log\;\text{Pr}\left(m_{uv}\right)}{\log\;\text{Pr}\left(u\right)+\log\;\text{Pr}\left(u\right)}\left(1-\text{Pr}\left(m_{uv}\right)\right). \end{array}$$

Jiang and Conrath [13] define the dissimilarity between two terms as

(11) $$\begin{array}{c} D_{uv}^{\left(\text{Jiang}\right)}=\log\;\text{Pr}\left(u\right)+\log\;\text{Pr}\left(u\right)-2\log\;\text{Pr}\left(m_{uv}\right). \end{array}$$

This is equivalent to defining its reciprocal as the similarity as

(12) $$\begin{array}{c} T_{uv}^{\left(\text{Jiang}\right)}=\frac{1}{\log\;\text{Pr}\left(u\right)+\log\;\text{Pr}\left(u\right)-2\log\;\text{Pr}\left(m_{uv}\right)}. \end{array}$$

The second group of approaches calculates similarity between GO terms depending on the structure of the gene ontology. Briefly, given a term indexed by *t*, Wang et al. iteratively calculate an *s*-value for every ancestor *a* ∈ *A*_*t*_ to measure the contribution of *a* to the semantic of *t* as

(13) $$\begin{array}{c} s_{t}\left(a\right)=\{\begin{array}{cc} 1 & \text{if}\;\;a=t,\\ \underset{x\in C_{a}}{\max}w_{e}s_{t}\left(x\right) & \text{if}\;\;a\neq t, \end{array} \end{array}$$

where the weight *w*_*e*_ = 0.8 if *x* and *t* have the is_a relationship and *w*_*e*_ = 0.6 if *x* and *t* have the part_of relationship [10]. Then, a semantic value for term *t* is defined as *s*(*t*) = Σ_*x*∈*A*_*t*__ *s*_*t*_(*x*). Finally, the semantic similarity score between two terms *u* and *v* is defined as

(14) $$\begin{array}{c} T_{uv}^{\left(\text{Wang}\right)}=\underset{x\in A_{u}\cap A_{v}}{\sum }\frac{s_{u}\left(x\right)+s_{v}\left(x\right)}{s\left(u\right)+s\left(v\right)}. \end{array}$$

With pairwise semantic similarity scores between GO terms being ready, the similarity between term *t* and a set of terms *T* is defined as

(15) $$\begin{array}{c} \text{Sim}\left(t,T\right)=\underset{t^{\prime }\in T}{\max}T_{tt^{\prime }}, \end{array}$$

where *T*_*tt*′_ is calculated using either of the above methods. The similarity between two sets of terms *S* and *T* can then be calculated as

(16) $$\begin{array}{c} \text{Sim}\left(S,T\right)=\frac{1}{\left|S\right|+\left|T\right|}\left(\underset{s\in S}{\sum }\text{Sim}\left(s,T\right)+\underset{t\in T}{\sum }\text{Sim}\left(t,S\right)\right). \end{array}$$

Finally, for two gene products *g* and *h* annotated by two sets of terms *G* and *H*, respectively, the semantic similarity between the two objects is then defined as

(17) $$\begin{array}{c} S_{gh}=\text{Sim}\left(G,H\right). \end{array}$$

## 5. Results

### 5.1. Data Sources

There have been quite a few domain specific ontologies available for characterizing entities in a variety of biological domains. Particularly, the OBO (open biological and biomedical ontologies) Foundry has released eight ontologies to provide standard descriptions of entities in biological domains [14]. Among these ontologies, biological process (BP), molecular function (MF), and cellular component (CC) are typically referred to as the gene ontology (GO), which has been widely used to describe functions of genes. The gene ontology also provides annotations of gene products for several well-studied model organisms, including yeast, fruit fly, and mouse [1]. In this paper, we focus on the biological process domain of GO and annotations of the budding yeast* Saccharomyces cerevisiae* to validate the effectiveness of the proposed measures. We extract 22,688 terms from the biological process domain of the gene ontology (released on April 27, 2012) and obtain 22,798 annotations of 6,383 yeast genes (released on April 28, 2012).

### 5.2. Distribution of Semantic Similarity Scores of Random Gene Pairs

**Figure 1:**
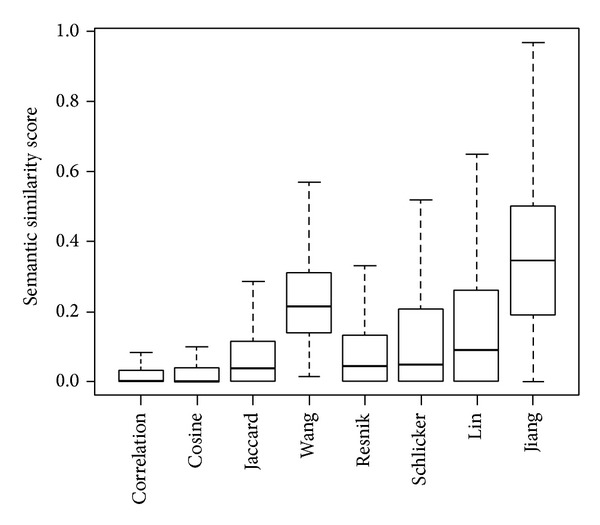
Distributions of semantic similarity scores of 100,000 randomly selected pairs of yeast genes.

It is evident that a pair of genes selected at random can hardly have similar functions, and thus the semantic similarity score between such a pair of genes should be close to zero. To validate this argument, we calculate semantic similarity scores of 100,000 pairs of yeast genes selected at random, and we summarize the distribution of the scores in Figure 1. We can clearly see from the figure that the median similarity score of the correlation measure (0.004894) is almost 0 so is that of the cosine measure (0.003196). The median similarity score of the Jaccard measure (0.03846) is higher than those for both the correlation and the cosine measures but still lower than those for all the five existing methods. The method of Resnik generates the smallest median similarity score (0.04395) among the existing methods, followed by the methods of Schlicker et al. (0.04810), Lin (0.09115), and Wang et al. (0.2138). The method of Jiang et al. generates the largest median similarity score (0.3460). From these observations, we conclude that the existing methods tend to overestimate semantic similarity between genes that are actually not related in their functions. On the other hand, the proposed measures, though much simpler than the existing methods, do not have such a drawback and thus yield much more reasonable results in assessing semantic similarity between randomly selected gene pairs.

### 5.3. Consistency between Gene Semantic Similarity and Pathway Data

It is known that most biological functions rise from collaborative effects of several proteins that usually involve in the same biological process and form a pathway [17]. Hence, gene products (proteins) in the same pathway should have similar annotations in the biological process ontology and in turn own high semantic similarity scores according this ontology. On the contrary, gene products belonging to different pathways should own relatively low semantic similarity scores. To assess whether the proposed similarity measures are consistent with this knowledge, we compare semantic similarity scores between proteins within a pathway and those between proteins involved in different pathways as follows.

**Figure 2:**
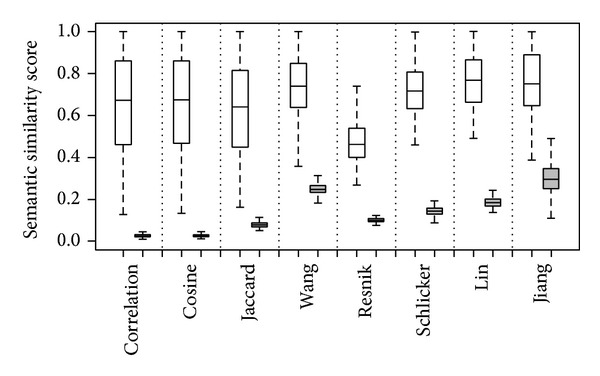
Distributions of mean semantic similarity scores within pathways (white) and outside pathways (gray).

We download from the* Saccharomyces* Genome database (SGD) [18] 141 pathways, each including at least two proteins. For each of these pathways, we calculate pairwise semantic similarity scores of proteins involved in the pathway, and we average these scores over all pairs of proteins to obtain the mean semantic similarity score within the pathway (*μ*_in_). Meanwhile, for each pathway, we further select at random 10 times the number of proteins as those in the pathway, calculate semantic similarity scores between these proteins and those in the pathway, and average over these scores to obtain the mean semantic similarity score outside the pathway (*μ*_out_). Then, we plot the distribution of mean similarity scores within and outside all pathways in Figure 2. From the figure, we observe that the mean similarity scores within pathways are in general large, while those outside pathways are typically small. Particularly, for all of the three proposed measures (correlation, cosine, and the Jaccard), the differences between the medians of the mean similarity scores within and outside pathways are much more obvious than those of the five existing methods. For example, using the correlation measure, we obtain the median *μ*_in_ over all pathways as 0.6578 and the median *μ*_out_ as 0.02564. Using the cosine measure, we obtain a median *μ*_in_ of 0.6600 and a median *μ*_out_ of 0.02733. In contrast, the method of Wang produces a median *μ*_in_ of 0.7405 and a median *μ*_out_ of 0.2489, and the method of Resnik produces a median *μ*_in_ of 0.4662 and a median *μ*_out_ of 0.09956.

**Figure 3:**
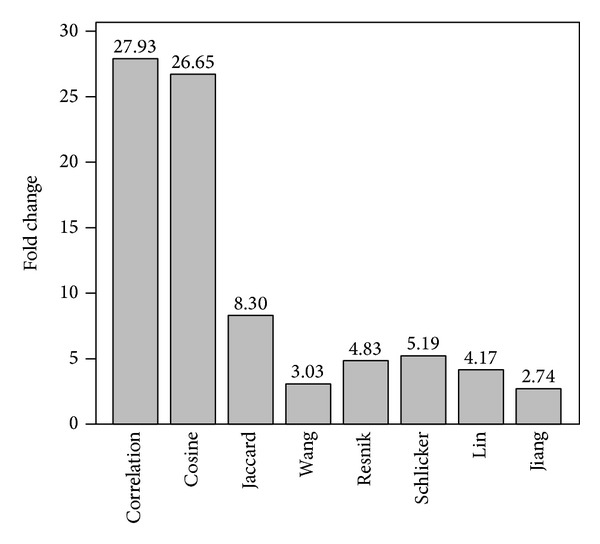
Fold change of semantic similarity scores within pathways against those outside pathways.

We further calculate for each pathway the ratio of the mean semantic similarity scores within the pathway over that outside the pathway (*μ*_in_/*μ*_out_), and we average such ratios over all 141 pathways to obtain a criterion called fold change of semantic similarity scores within pathways against those outside pathways. We summarize the fold changes in Figure 3, from which we can clearly see the effectiveness of the proposed measures. For example, using the correlation measure, we obtain a fold enhancement of 29.93. Using the cosine measure, we obtain a fold change of 26.65. In contrast, the method of Wang only produces a fold change of 3.03, and the method of Resnik produces a slightly larger fold change of 4.83.

These observations support the conclusion that the proposed measures yield much more reasonable results in assessing functional relationships between proteins within pathways, and thus these measures are more consistent with biological knowledge than existing methods.

### 5.4. Consistency between Gene Semantic Similarity and Protein Domain Data

Proteins are often composed of one or more functional regions, commonly referred to as protein domains [19]. Different domains typically account for different functions of proteins containing them, and thus different combinations of protein domains give rise to the diverse range of proteins found in nature. Hence, proteins can be classified into different families according to the domains that the proteins contain. Moreover, proteins containing the same domain, or say belonging to the same family, should have some similar functions and thus share some similar annotations in the biological domain of the gene ontology. Consequently, proteins belonging to the same family should have high semantic similarity scores according to the gene ontology. On the contrary, proteins belonging to different familiess should own relatively low semantic similarity scores. To assess whether the proposed similarity measures are consistent with this knowledge, we compare semantic similarity scores between proteins within a protein family and those between proteins belonging to different families as follows.

**Figure 4:**
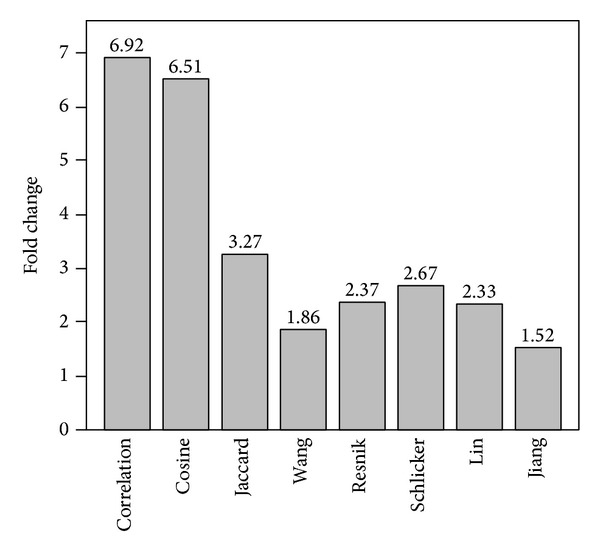
Fold change of semantic similarity scores within protein families against those outside protein families.

The Pfam database [20] provides a large collection of both high quality protein families (Pfam-A) and low quality protein families (Pfam-B). In version 26.0 of the Pfam-A collection (released in November 2011), 13,672 protein families are collected. From this data source, we extract 1,022 protein families, each including at least two yeast proteins. For each of these families, we calculate pairwise semantic similarity scores of proteins belonging to the family, and we average these scores over all pairs of proteins to obtain the mean semantic similarity score within the family (*ν*_in_). Meanwhile, for each protein family, we further select at random 10 times the number of proteins as those in the family, calculate semantic similarity scores between these proteins and those belonging to the family, and average over these scores to obtain the mean semantic similarity score outside the family (*ν*_out_). Then, we calculate for each protein family the ratio of the mean semantic similarity scores within the family over that outside the family (*ν*_in_/*ν*_out_), and we average such ratios over all 1,022 protein families to obtain a criterion called fold change of semantic similarity scores within protein families against those outside families. We summarize the fold changes in Figure 4, from which we can clearly see the effectiveness of the proposed measures. For example, using the correlation measure, we obtain a fold change of 6.915. Using the cosine measure, we obtain a fold change of 6.511. Using the Jaccard measure, we obtain a fold change of 3.267. In contrast, the method of Wang only produces a fold change of 1.856, and the method of Resnik produces a slightly larger fold change of 2.370.

We further change the minimum number proteins belonging to a protein family from 2 to 10, calculate the fold change in each situation, and present the results in Table 1. Briefly, the fold change varies with the minimum number of proteins in a protein family, but the observation that the fold changes of the proposed measures are greater than those of the existing methods remains unchanged. For example, when considering protein families containing at least 10 proteins, we obtain fold changes of 9.273, 9.814, and 4.516 for the correlation, cosine, and the Jaccard measures, respectively. In contrast, the fold change for the measures of Wang, Resnik, and Schlicker are 2.090, 2.846, and 3.430, respectively. From these results, we make the conjecture that the proposed measures yield much more reasonable results in assessing functional relationships between proteins that belong to the same protein family. Hence, we conclude that the proposed measures are more consistent with biological knowledgethan existing methods.

### 5.5. Consistency between Gene Semantic Similarity and PPI Data

Biological knowledge suggests that proteins often interact with each other in the collaborative generation of biological functions [21]. The collection of all physical interactions in a living organism is typically referred to as the protein-protein interaction (PPI) network, in which nodes are proteins and edges are physical interactions between the proteins. Interacting proteins are usually involved in similar biological process and thus have similar annotations in the biological process domain of the gene ontology and high semantic similarity scores. To assess whether our similarity measures are consistent with this knowledge, we assess relationships between interacting proteins and their semantic similarity scores as follows.

**Figure 5:**
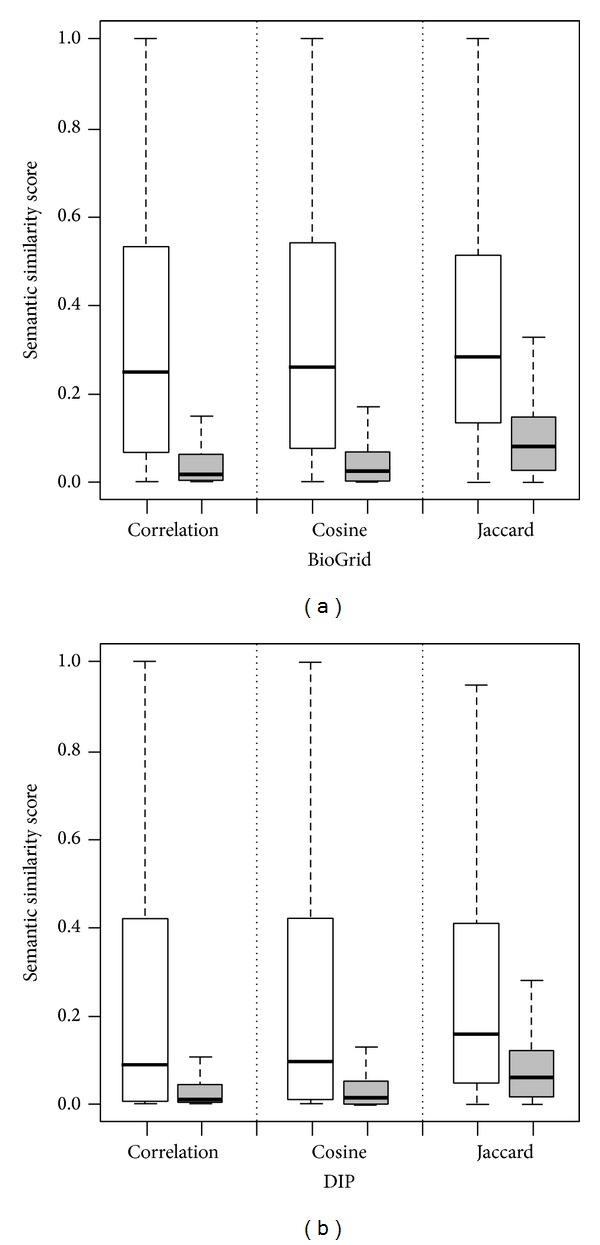
Relationships between semantic similarity scores and protein-protein interaction data. (a) Distributions of similarity scores of interacting proteins (white) against noninteracting proteins (gray) for the BioGrid dataset. (b) Distributions of similarity scores of interacting proteins (white) against noninteracting proteins (gray) for the DIP dataset.

We download two manually curated PPI networks of* Saccharomyces cerevisiae*. From BioGrid (biological generic repository for interaction datasets) [22, 23], we extract a PPI network composed of 3,529 nodes and 16,285 edges. From DIP (database of interacting proteins) [24, 25], we extract a relative small PPI network including 2,902 nodes and 7,005 edges. For each of these networks, we calculate semantic similarity scores for interacting proteins and those for the same number of randomly selected noninteracting pairs of proteins, and we plot the distribution of these scores in Figures 5(a) and 5(b). From the figure, we obviously see that the semantic similarity scores for interacting proteins are in general larger than those for noninteracting proteins, and this observation exists for both the BioGrid and the DIP networks.

**Figure 6:**
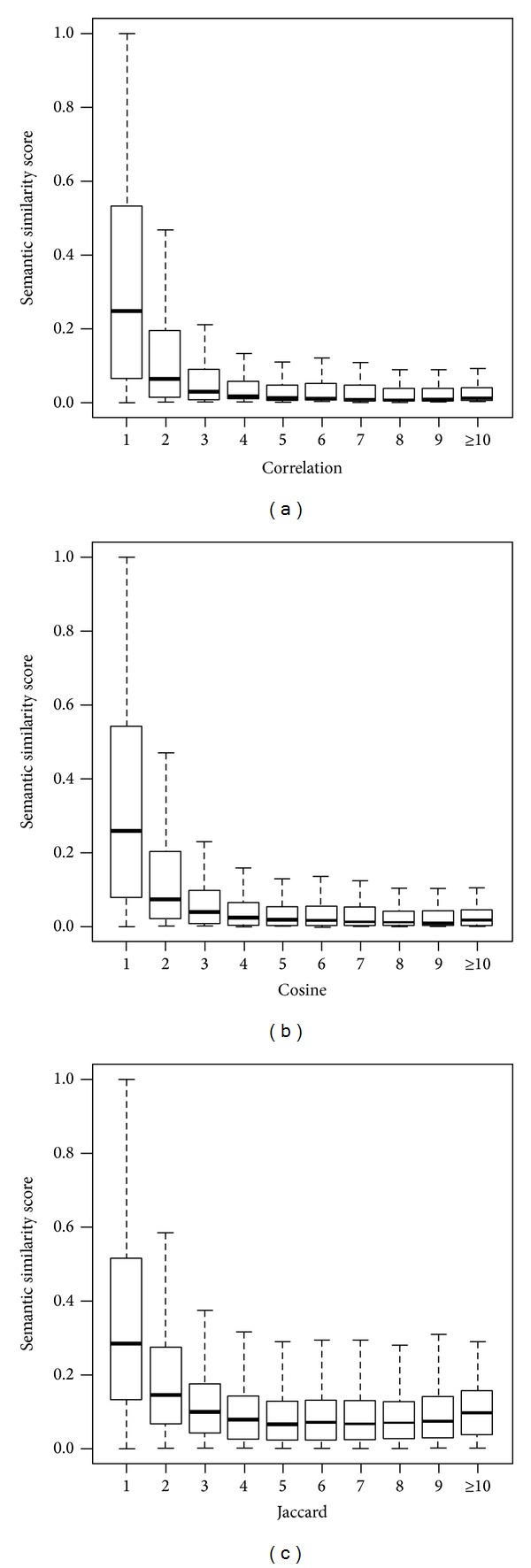
Distributions of semantic similarity scores against the shortest path distance of interacting proteins for the BioGrid dataset. (a) Results for the measure of correlation. (b) Results for the measure of cosine. (c) Results for the measure of Jaccard.

Then, for each of these networks, we average over semantic similarity scores between interacting proteins to obtain the mean semantic similarity score of interacting proteins (*τ*_int⁡_). Meanwhile, we average over semantic similarity scores of noninteracting pairs of proteins to obtain the mean semantic similarity score of noninteracting proteins (*τ*_non_). Finally, we calculate the fold change as *τ*_int⁡_/*τ*_non_ to measure the effectiveness of a method in distinguishing the functional relationship between interacting proteins. We present the results summarized in Figure 6, from which we can see the effectiveness of the proposed measures. For example, for the BioGrid network, we obtain a fold change of 6.15 when using the correlation measure. For the DIP network, the fold change is 5.44 for the correlation measure. For the cosine and the Jaccard measures, we observe similar results. From these observations, we make the conjecture that the semantic similarity scores calculated by the proposed measures are consistent with biological knowledge about interacting proteins.

It has also been shown that proteins closer in a PPI network tend to have more similar functions [4]. With this understanding, we use the length of the shortest path between two proteins in a PPI network to measure the network proximity of the proteins, use the semantic similarity score of the two proteins to measure their functional similarity, and plot the change of the similarity score with the closeness of proteins in Figure 6. From the figure, we can see that protein pairs tend to have higher semantic similarity scores if they are closer in the PPI network. For example, for the BioGrid network and the cosine measure, the median semantic similarity score is 0.2590 for direct interacting protein pairs, 0.0720 for protein pairs intermediated by another protein, 0.0372 for protein pairs intermediated by two other proteins, and so forth. Similar results are observed for the other two measures. These results suggest that protein similarity scores are correlated with protein closeness in a PPI network, again consistent with biological knowledge.

## 6. Conclusions and Discussion

In this paper, we have proposed an approach to represent annotations of a gene product in the gene ontology using vectors that are composed of information contents of terms in the ontology. Based on this notion, we have proposed to calculate pairwise semantic similarity between gene products by using three measures (Pearson's correlation coefficient, cosine similarity, and the Jaccard index) to quantify the relatedness of the corresponding vectors. We have performed a series of comprehensive studies on the effectiveness of the proposed measures using the ontology of biological process and annotations of the budding yeast* Saccharomyces cerevisiae*. Comprehensive studies on the relationships between semantic similarity of gene products and biochemical pathways, protein families, and protein-protein interaction networks show that semantic similarity scores calculated using the proposed measures are more consistent with biological knowledge than those derived using a list of five existing methods, suggesting the effectiveness of our method in characterizing functional similarity between gene products based on the gene ontology.

The main advantage of the proposed measures is the simplicity in calculation and the effectiveness in characterizing semantic similarity between gene products. The representation of gene products as vectors of information contents of ontology terms is straightforward, making the followed computation easy to understand. The simplicity in presentation also benefits the computation with a low time complexity, thereby making our method suitable for large scale calculation of semantic similarity for not only applications based on the gene ontology but also those using other ontologies.

Certainly, the proposed measures can be further improved from the following aspects. First, although the contribution of a term in a domain ontology has been characterized by its information content, it is possible to further refine such contribution by adjusting the information contents with prior knowledge. For example, it is not hard to combine annotations of different organisms to achieve a more precise estimation of information contents for concepts in the gene ontology. Another possibility is to develop a Bayesian method to estimate the information contents, using existing annotations to derive the prior distribution.

Second, although the presentation of domain entities as vectors of concepts is simple yet effective, the incorporation of the structure of the concepts in the underlying ontology may further improve the performance of the proposed method. Existing algorithms for calculating similarity between two tree structures [26] might be a potential candidate along this direction.

## Figures and Tables

**Table 1:** Fold changes of semantic similarity scores within protein families against those outside families.

*m*	*n*	Semantic similarity measures							
		Correlation	Cosine	Jaccard	Wang	Resnik	Schlicker	Lin	Jiang
2	1022	6.915	6.511	3.267	1.856	2.370	2.669	2.331	1.524
3	562	8.986	8.446	3.827	1.988	2.680	3.100	2.641	1.629
4	360	9.608	8.760	4.027	2.037	2.799	3.247	2.761	1.656
5	240	9.359	9.135	4.131	2.065	2.843	3.324	2.827	1.662
6	182	9.997	9.214	4.224	2.105	2.901	3.410	2.888	1.692
7	141	10.10	9.741	4.363	2.106	2.952	3.476	2.918	1.690
8	110	9.921	9.409	4.432	2.101	2.853	3.409	2.895	1.661
9	89	9.880	9.321	4.445	2.094	2.857	3.419	2.908	1.643
10	75	9.814	9.273	4.516	2.090	2.846	3.430	2.898	1.644

*m*: minimum number of proteins in a family. *n*: number of protein families, each containing at least *m* proteins.
